# Embedded real-time analysis of continuous casting for machine-supported quality optimisation

**DOI:** 10.12688/openreseurope.20547.1

**Published:** 2025-08-08

**Authors:** Kersten Marx, Yalçın Kaymak, Zeinab Kargar, Robin Jentner, Nico Neuber

**Affiliations:** 1 Process Optimisation Iron and Steel Making, VDEh-Betriebsforschungsinstitut GmbH, Düsseldorf, North Rhine-Westphalia, 40489, Germany; 2 Quality and Information Technologies, VDEh-Betriebsforschungsinstitut GmbH, Düsseldorf, North Rhine-Westphalia, 40489, Germany; 3 Stahlwerk – Technologie und Forschung, AG der Dillinger Hüttenwerke, Dillingen/Saar, Saarland, 66763, Germany

**Keywords:** Continuous Casting, Big Data, Digital Twin, Computational Fluid Dynamics

## Abstract

**Background:**

Thermal and fluid-mechanical conditions in continuous casting (CC) moulds are only roughly known although highly relevant for the product quality. Manual process control is difficult due to the big number of influencing factors. During continuous casting, manual top-freezing controls must be carried out. Every manual performed mould control can affect the strand quality and even increase the risk of failure. Therefore, regular top-freezing controls are performed after a certain casting duration. However, top-freezing events between the regular controls cannot be detected and are a major risk for plant safety.

**Methods:**

In the RFCS project RealTimeCastSupport, the aim of the research was the digitalisation and optimised control of continuous casting machines. A real-time support system was developed to predict quality-relevant top-freezing events and thus achieve improved control. This was reached by offline material tracking, synchronisation of data streams and statistical analysis by application of Big Data technologies, the development of a digital twin and the exploitation of various CC data and surface inspection to predict reliability of steel production.

**Results:**

The following results were achieved:

- Identification of defect promoting scenarios by correlation of statistical results and surface defect detection.
- Realisation of an offline 3D digital twin of the mould considering heat transfer, inert gas feeding and solidification.
- Offline reproduction of the identified defect promoting scenarios with the 3D digital twin to find thermal and fluid mechanical reasons for the detected behaviour.

**Conclusions:**

The application of a real-time support system enables the prediction of top-freezing events during the whole casting process. Subsequently, this significantly increases the plant safety and offers to carry out top-freezing inspections in a more targeted manner in the future.

This publication is part of a series of papers in the frame of the dissemination project METACAST.

## Objectives

Thermal and fluid-mechanical conditions in continuous casting (CC) moulds are only roughly known although highly relevant for the product quality. Manual process control is difficult due to the big number of influencing factors. Therefore, the aim of the RFCS research project RealTimeCastSupport was the digitalisation and optimised control of continuous casting machines. An important objective was to predict quality-relevant top-freezing events and thus achieve improved control.

This is reached by exploitation of various CC data and surface inspection to predict reliability of steel production:

- Offline material tracking, synchronisation of data streams and statistical analysis by application of Big Data technologies.
- Identification of defect promoting scenarios by correlation of statistical results and surface defect detection.
- Realisation of an offline 3D digital twin of the mould considering heat transfer, inert gas feeding and solidification.
- Offline reproduction of the identified defect promoting scenarios with the 3D digital twin in order to find thermal and fluid mechanical reasons for the detected behaviour.

## Methods

### Data handling for the real-time support system

Top-freezing is an unwanted event that can cause surface defects or even lead to breakouts in continuous casting. A real-time support system was developed to reduce these defects. The information gained by the implemented measurement systems at AG der Dillinger Hüttenwerke (AGDH) was therefore transferred to VDEh-Betriebsforschungsinstitut GmbH (BFI) for the development and test phase of a real-time support system. This was done by the installation of a message broker system (Eclipse Mosquitto) at AGDH and BFI. The message broker realises the delivery of data from AGDH to BFI by a secured internet connection.

Additionally, the data packages were encrypted during their transport, so all necessary security requirements were fulfilled. The data from AGDH was stored in a NoSQL database (MongoDB). Those databases are structured by objects and are more flexible in the types of stored information as conventional database systems. Just the temporary integration of measurements and the integration of new data sources is supported by NoSQL database schemes.

The system parts installed at BFI were integrated into Virtual Machines (VM) to simplify the transfer and the integration of those parts to AGDH after the test and development phase.

### Assignment to the corresponding casting conditions at AGDH

**Figure 1.**
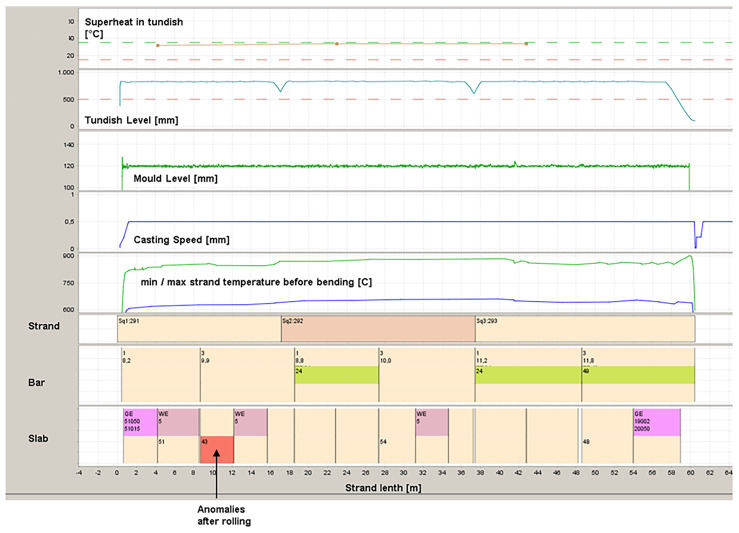
Assignment of casting conditions and results of rolling process.

Since every heavy plate is tailor-made and therefore an individual construction, it has always been necessary at AGDH to match the results of the rolling with those of the casting process to enable continuous process development (see Figure 1).

This means that the material tracking from the strand to the slab that is then rolled is completely stored in the AGDH database systems and is available for further investigations. In the event of any anomalies in the finished rolled plates, a backwards allocation is carried out and the corresponding slabs are identified. This enables easy assignment to the casting parameters. Since the Eddy Current (EC) system is fully integrated into the AGDH quality systems, the results of the surface defect detection are also mapped in this way.

AGDH already provides time stamps in the data sets for “top surface freezing in the mould” as a part of the real-time data transmission to BFI. This information can be used as a model input. If the occurrence of surface defects is still too rare for model development, it can also be used as a modelling target until a higher amount of data with surface defects in the data set is available. This is justifiable because internal research suggests that these two events are related anyway, but AGDH does not yet have a satisfactory avoidance strategy for this event, too.

### Layout, design and commissioning of the Big Data environment

First, all available information on the CC process was collected and reviewed. More than 40 different parameters were identified for evaluation. These are on the one hand general information about the respective melt or plate and on the other hand time series of different resolution.

To simplify data handling, the most flexible architecture possible and the most intuitive tools possible were selected. For easy linking of data from different data sources the programming tool Node-Red was used. It provides a browser-based editor that makes it easy to wire together flows using the wide range of nodes in the palette that can be deployed to its runtime in a single-click. To make all data available in the steel plant, the object-relational database management system PostgreSQL with the extension for time series TimescaleDB was chosen as the Big Data environment. This is particularly necessary for the increased data volume during the acquisition of the mould level measurement in 50 ms frequency.

To make all data available to BFI, a real-time data transfer was realized via a Message Queuing Telemetry Transport (MQTT) protocol. The data is secured via a Python implementation of end-to-end encryption as a service via MQTT.

In order to synchronize the time series from the various systems of the steel mill and the new measuring equipment from the developments in this project, a system time of the Big Data environment was defined in addition to the local time of the respective measuring equipment. In the system time of the Big Data environment, a time stamp is generated for each piece of information in a time series when the data is saved. Since this saving happens in real time, there is a high accuracy in the data synchronization.

For the first offline data analytics AGDH provided to BFI data sets of five different charges (with 67 different measurement parameters), two of them include surface defects. As a reminder, one possible cause for the formation of surface defects is the possible "freezing" of melted casting powder in the area of the meniscus at the strand shell. For this reason, BFI focused on the heat flow densities [MW/m ^2^], tundish weight [t] and the mould fill level [mm].

## Results

### Identification of defect promoting scenarios

It became clear that without sophisticated analysis methods, it will be difficult to identify the influencing factors for the target parameters for defect promoting scenarios. Therefore, several procedures were executed to find differences between process intervals with the occurrence of surface defects and without:

- using time/position based sliding windows for calculating features derived from the window data to extract different types of information regarding absolute amplitude (e.g. by mean, maximum, …), occurring variation (e.g. by standard deviation, signal range, …) and others
- applying methods of data analytics for feature extraction like Autoencoders which process complete signal curves at once in a deep learning approach
- using data mining methodologies for clustering the signals and/or derived features, aiming to find clusters related to higher risks of surface defect occurrence.

For this task, the data was transferred from the AGDH Data Warehouse to BFI and stored in the MongoDB. The data includes the control of top-freezing events information, signal data and information about the casting process. To use such data for the development of models to predict the events of interest, the data needs to be pre-processed to yield a reliable data set and additionally, it requires further selection of the most informative process variables for the phenomena under consideration.

Data preprocessing involves a few steps, which are: Labeling the data, selecting relevant data, creating windows around each event, splitting the data into a training set and a test set, and normalizing the data.

Top-freezing events are marked by start and end codes. Ideally, each start should have a corresponding end, but mismatches occur in the dataset. If an end code is missing, the event is assumed to last until the end of the casting sequence.

**Figure 2.**
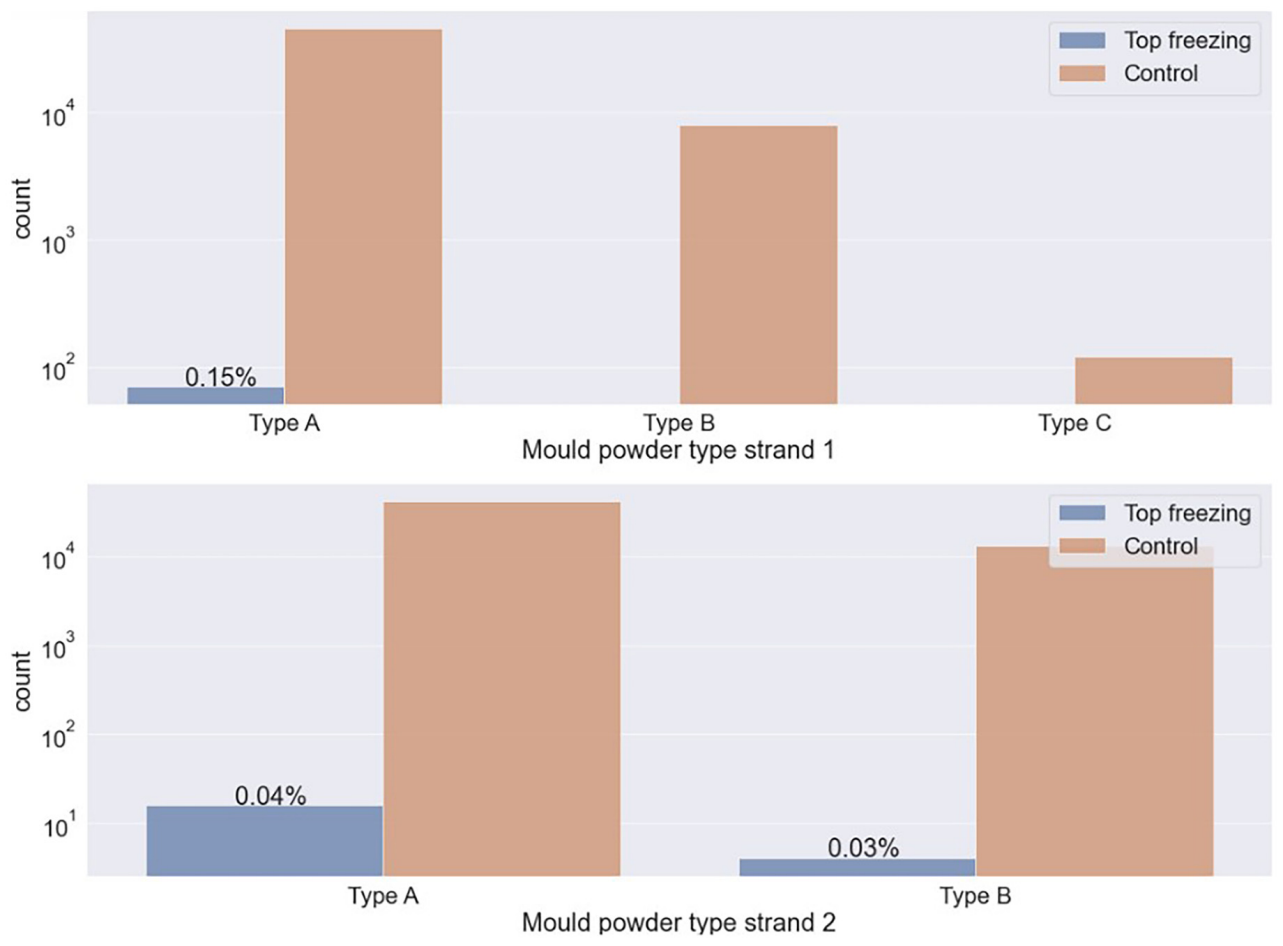
Percentage of top-freezing events in plants depending on mould powder type.

The data analysis of the mould powder type is shown in Figure 2. Type A has a lower carbon content than other types. The top-freezing events occur more frequently when using the mould powder type with lower carbon content. The carbon content of the mould powder type influences the solidification rate of the melt. This is because the melting time of the mould powder shortens with increasing carbon content in the mould powder. As a result, the melting rate increases, which leads more quickly to a protective slag layer against thermal insulation and minimizes the risk of top-freezing.

**Figure 3.**
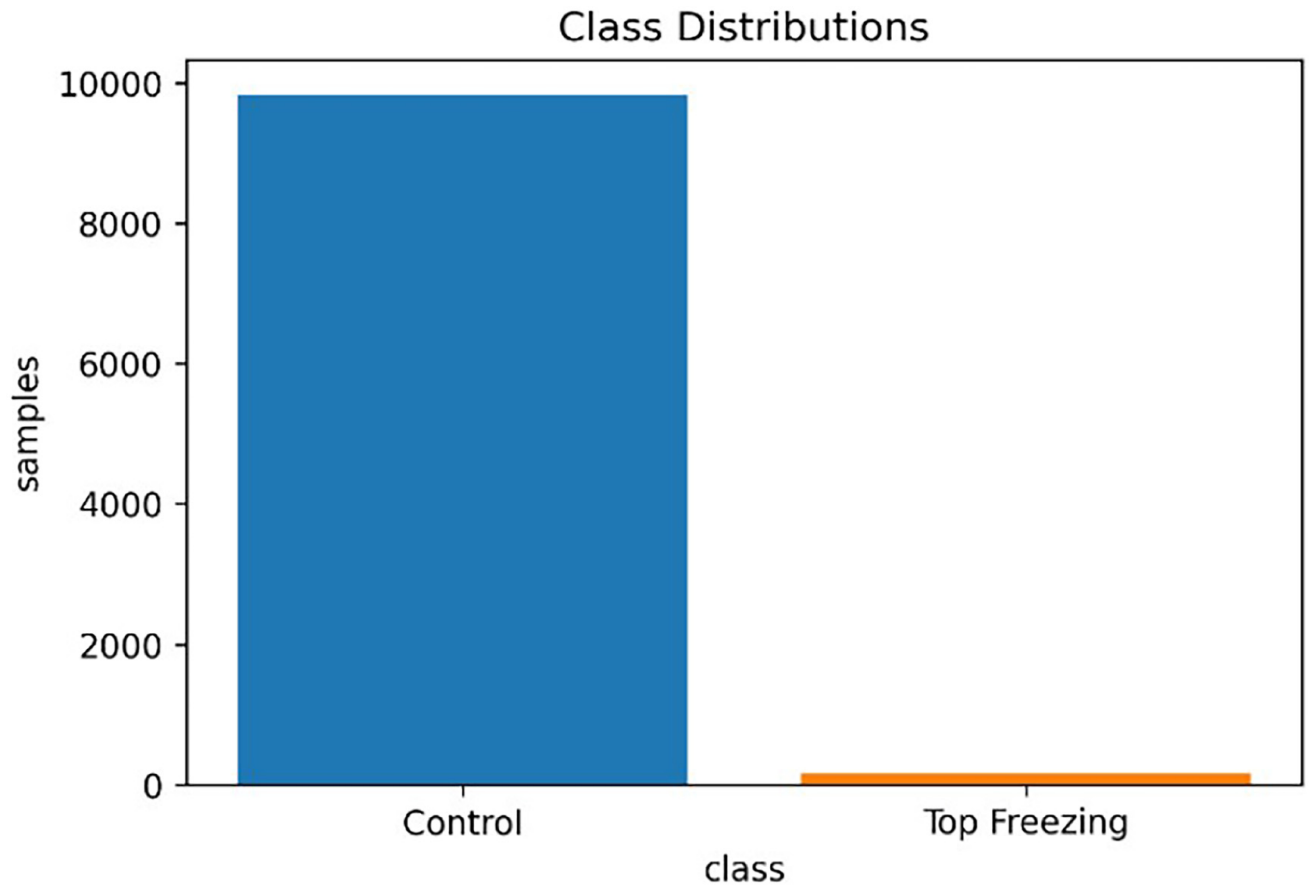
The data imbalance in dataset.

As shown in Figure 3 the observation of control events is higher (98.3%) than the observation of top-freezing events (1.6%). then this dataset is highly imbalanced which brings some challenges to it.

A subfield of machine learning that deals with this problem is called One-Class classification. This involves unsupervised learning algorithms that use a normal training dataset to create a model that represents normal behavior. Anomalies can then be detected by deviating from this model. One-Class Support Vector Machine (SVM), Isolation Forest and Autoencoder algorithms are used for anomaly detection.

**Figure 4.**
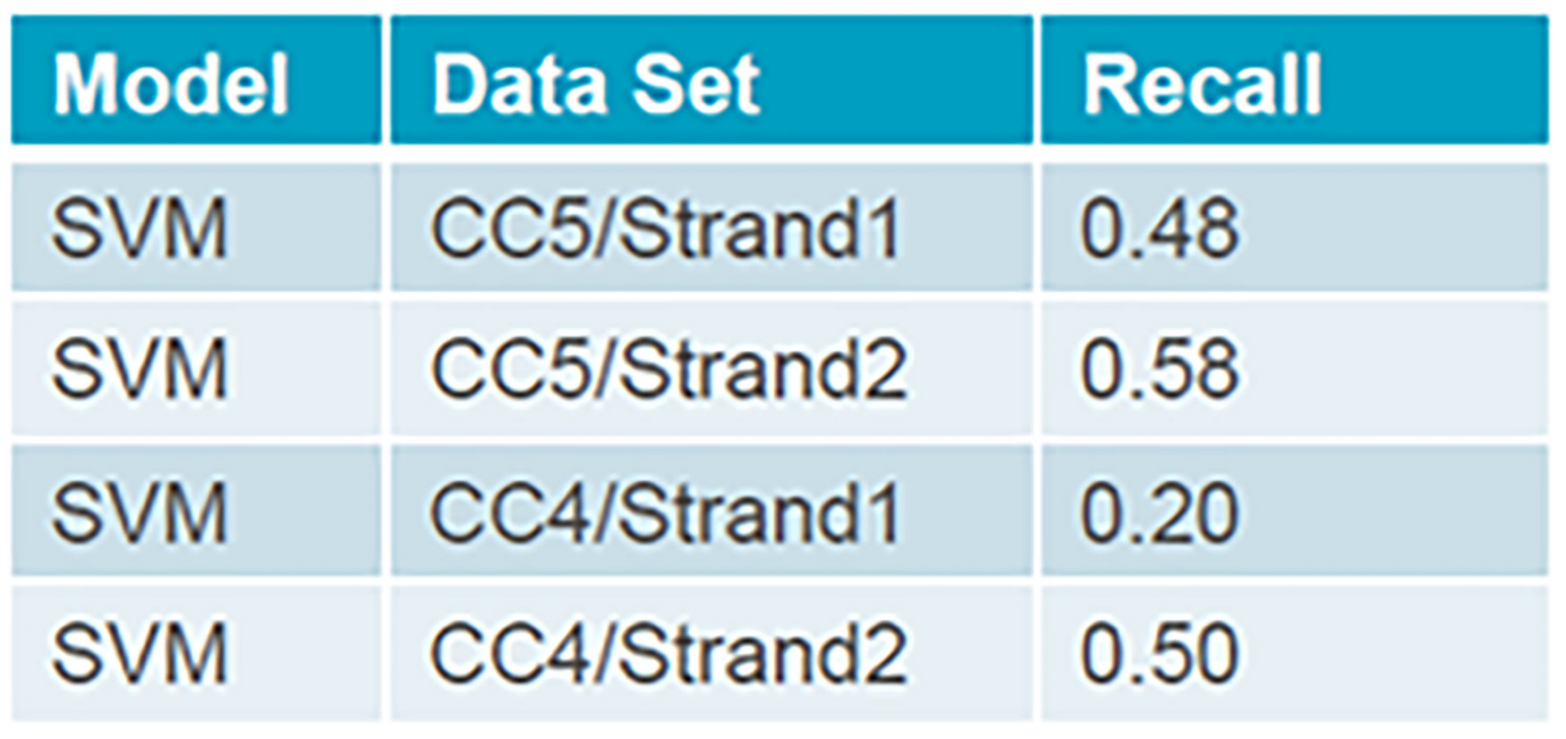
Recall metric for CC4 and CC5 to detect top-freezing events using One-Class SVM.

After training a machine learning model, it is important to know the effectiveness of the model. The better the effectiveness, the better the performance. To evaluate the model for highly imbalanced datasets, the recall metric is used. Recall shows what proportion of the top-freezing events were identified correctly for the continuous casting machines CC4 and CC5, Figure 4.

Tuning the hyperparameters is an essential part of controlling the behavior of a machine learning model. Depending on how costly it would be to make false-positive or false-negative classifications, it can be tuned to detect all top-freezing events.

### Setup of a digital twin of the casting machines based on CFD modelling

CFD is a recommended tool to simulate continuous casting and is used at BFI for many years ^1– 12^. Two different casting sizes and two different submerged entry nozzle (SEN) coverage heights have been computed for the AGDH caster using Ansys/Fluent software. Various process parameters were varied, and the process sensitivity was analyzed. In these models, the casting powder is modelled as an insulation layer with given thickness (45 mm) on top of the melt. The copper mould is explicitly included in the model so that the mould heat transfer is also resolved. The secondary cooling below as well as the heat losses at top surface approximated by an equivalent convective cooling. The inert gas injection at the SEN is modelled by using the DPM (discrete phase model) available in Ansys/Fluent software. The computational fluid dynamics (CFD) model focuses on the solidification of the liquid steel (i.e., neither powder melting nor slag solidification). The model mainly computes the flow field and temperature field. This also means shell thickness and detailed heat flux within the mould. Currently, the model neither computes stress & deformation nor predicts surface cracks or defects.

In order to investigate the effects of port coverage, SEN geometry, and casting size on the flow field in mould, the steel shell formation, the heat-fluxes in the mould, and top-freezing phenomena, 4 computations are performed:

- Case1: SEN-A, 80 mm port coverage, 400x2200 format
- Case2: SEN-B, 80 mm port coverage, 400x2200 format
- Case3: SEN-B, 120 mm port coverage, 400x2200 format
- Case4: SEN-B, 120 mm port coverage, 600x2200 format

**Figure 5.**
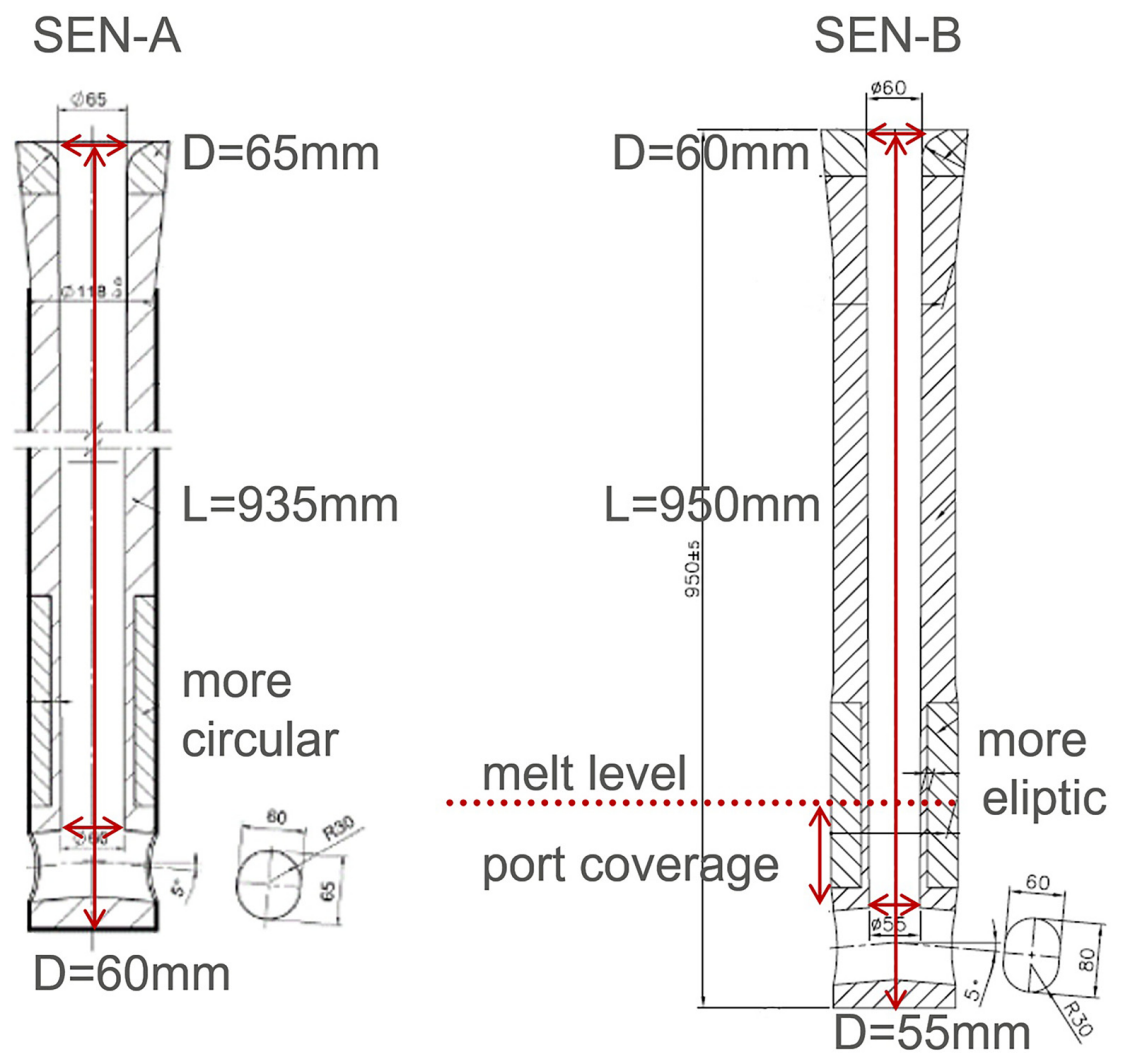
The geometry of SEN-A and SEN-B.

**Figure 6.**
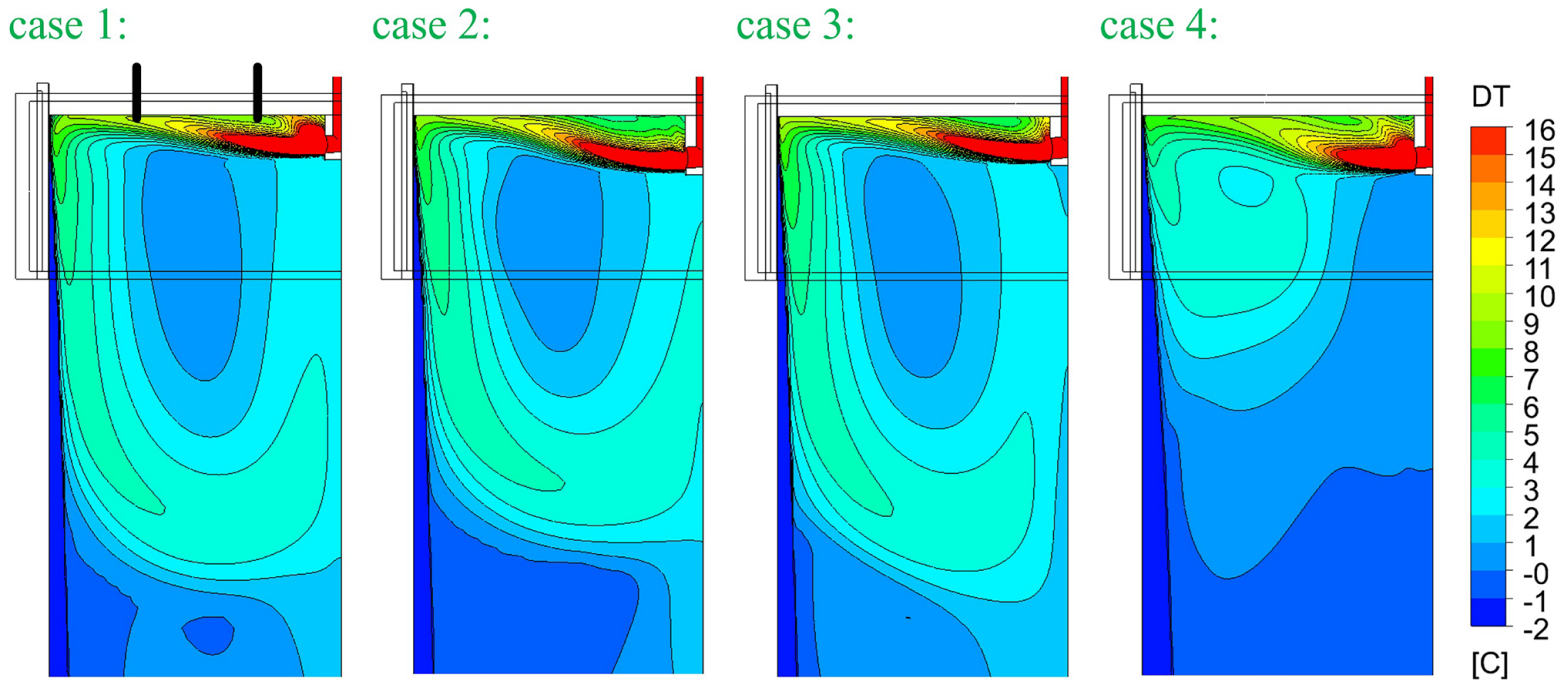
Comparison of temperature fields.

**Figure 7.**
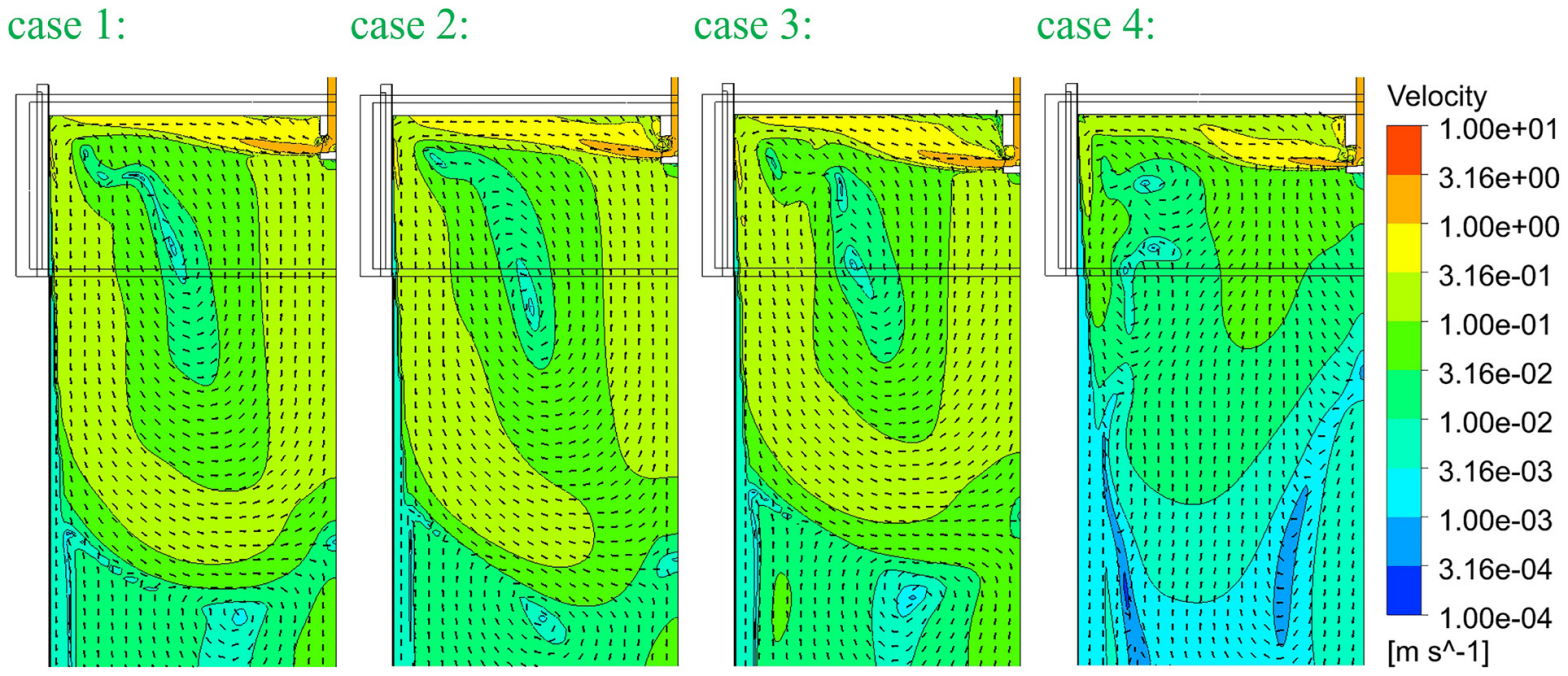
Comparison of the flow velocities.

The geometry of SEN-A and SEN-B are briefly shown in Figure 5. In principle, SEN-A has a smaller outflow port and SEN-B has a smaller inner diameter. The computed temperature and flow fields are compared in Figure 6 and Figure 7, respectively. The temperature and flow field in case 4 seem to be different. This is mainly due to the reduced casting speed. Although the cross-section is 1.5 times larger, casting speed is 0.5 times smaller. So, the velocity at SEN port is ca. 0.75 times smaller. This reduced momentum and consequently changed the flow and temperature fields.

**Figure 8.**
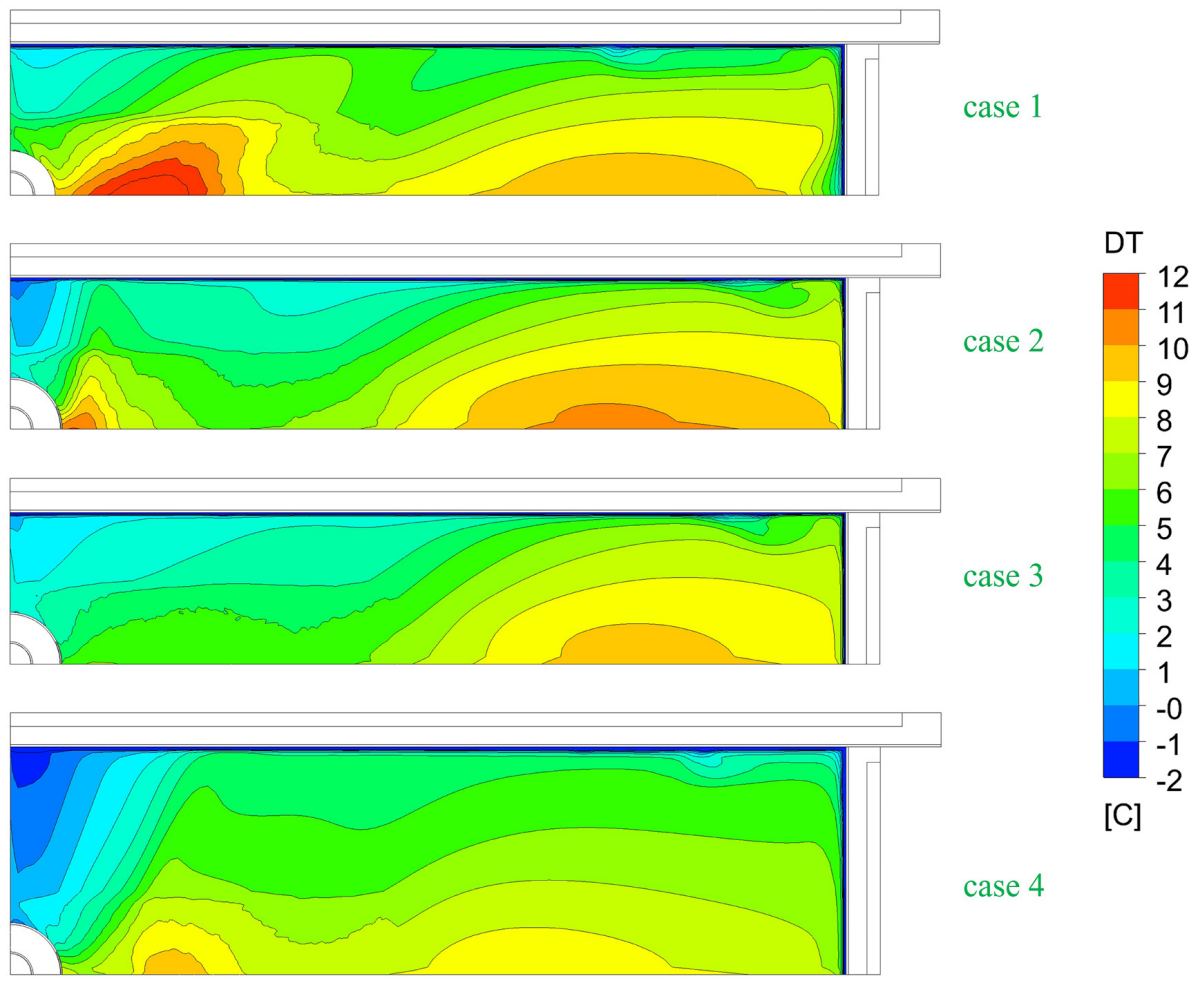
Comparison of the average steel melt temperatures at the top surface.

The top-freezing phenomena is somewhat related to the steel melt temperature and turbulence at the top surface. To judge the danger of top-freezing, the average steel melt temperatures are plotted in Figure 8. For the larger port coverages, top-freezing danger slightly increases! For the larger format, top-freezing danger even further increases due to reduced SEN-jet speed.

The sensitivity of the following model outputs (right column) to process input parameters (left column) is analyzed:

**Table:** 

• inlet super heat: DTin • casting speed: v_cast • melt viscosity, mu • casting powder layer thickness, tcp • thermal res. layer narrow face, trl_n • thermal res. layer wide face, trl_w • Fluent parameter, mushy zone const., mzp • Ar injection rate, Var	• average heat flux at narrow face: qn • average heat flux at wide face: qw • narrow side average shell thickness • wide side average shell thickness • average top surface melt temperature • average velocity at top surface • average turbulence at top surface

**Figure 9.**
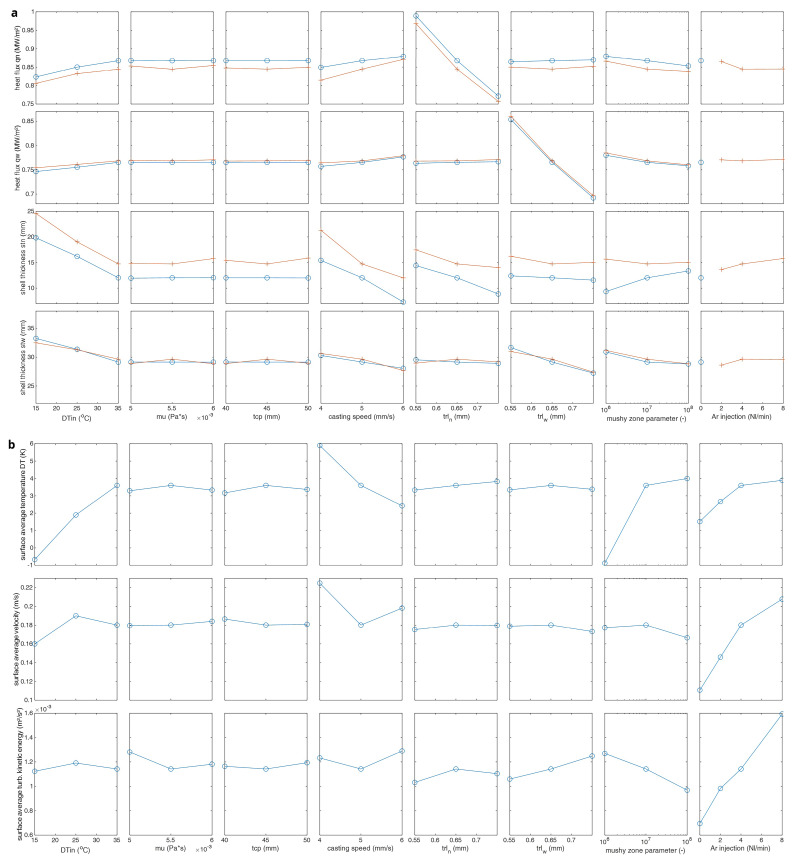
The sensitivity matrix.

The sensitivity matrix is plotted in Figure 9. In the upper matrix, two curves are plotted. The red curve shows results with 4 Nl/s argon (Ar) injection and blue curve no argon injection. If the super heat DTin increases, the heat fluxes to the mould also increase but the shell thickness at the mould exit decreases since the solidification takes longer due to higher temperature. The average steel melt temperature at top surface also increases and top-freezing danger is reduced.

There is no clear influence of the steel melt viscosity and the thickness of the casting powder for the investigated parameter interval. The casting speed is one of the main casting parameters. For higher casting speeds, the steel melt has less time for solidification. So, the steel shell thickness reduces (i.e., over all thermal resistance also reduces) and the heat flux to the mould increases.

The thermal resistive layer between the steel melt and the mould directly influences the heat flux. So, thicker thermal resistive layer means less heat flux and less steel shell formation. The built-in mushy zone parameter in Ansys/Fluent software controls the momentum sink in the mushy zone (i.e., dendrite size or permeability of porous region). It has a clear influence, but it has not been experimentally measured in this study. Its value is assumed to be mzp=10 ^7^ as suggested in the Ansys/Fluent user manual.

The argon injection (or inert gas injection) is also one of the main casting parameters. The jet stream from the SEN is bent upwards by the buoyancy of the gas bubbles. So, increased injection rates mean increased average steel melt temperature at the top as well as increased turbulence. This also reduces the top-freezing danger. In conclusion, there are 3 process parameters which have a clear influence on the top-freezing phenomena: melt super heat DTin, casting speed and inert gas injection rate.

**Figure 10.**
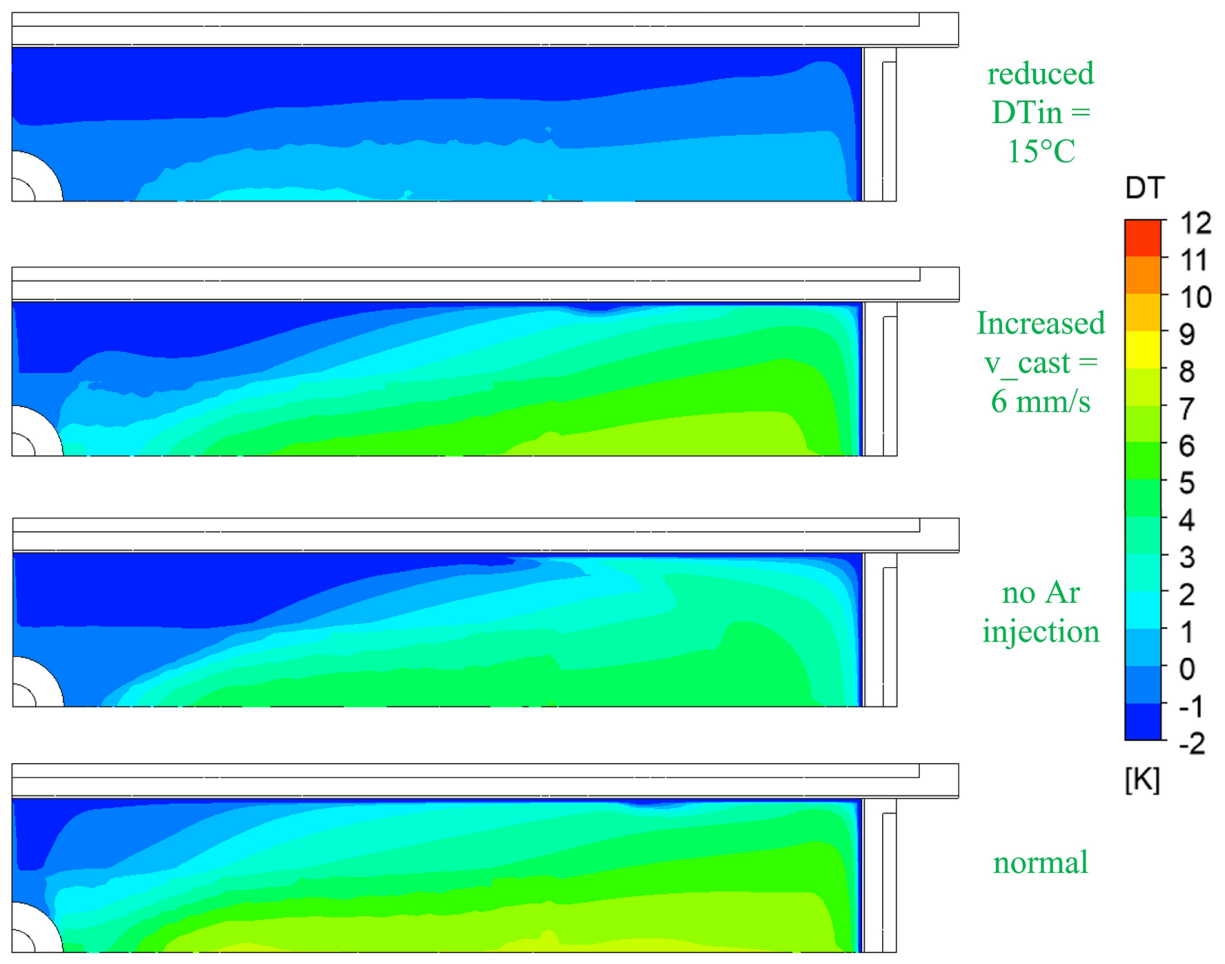
Comparison of melt temperature at the top for top-freezing promoting cases.

Three process parameters, namely, the melt super heat DTin, casting speed and inert gas injection rate are identified to have a clear influence on the top-freezing phenomena in the parameter sensitivity analysis. The results of these top-freezing promoting cases are further discussed here. Figure 10 shows the comparison of the melt temperature at the top surface for normal and top-freezing promoting cases (i.e., reduced inlet super heats DTin, increased casting speeds v_cast, and no Ar injection).

The most influencing factor for top-freezing is the super heat of the melt at SEN inlet as seen in Figure 10. When the inlet temperature is lower, the temperature at the top surface rapidly approaches the solidification temperature. Hence, this promotes the top-freezing incidences.

The melt super heat, casting speed and inert gas injection rate are identified to have a clear influence on the top-freezing phenomena. To avoid the top-freezing in the process, the inlet super heats DTin (i.e. the temperature in tundish) should be increased, the casting speeds v_cast should be decreased and, and inert gas injection should be increased. These counter-measures will tend to increase the melt temperature at the top surface in mould and thus help reduce the top-freezing.

### Implementation of a real-time support system for improved process control technology

Increasing the melt temperature in the tundish helps reduce the top-freezing. This can be achieved, i.e., by an increased melt temperature in the next ladle as long as the tundish has some space to add. Additionally, reducing the casting speeds and increasing the inert gas injection rates also help reduce the top-freezing.

It is about conducting industrial trials, an important phase in which we integrate the real-time support system into practical application. The software BFI developed has been successfully handed over to AGDH. Currently, a dedicated team of experts from AGDH is actively working on the online use of the software. Their main goal is to use the capabilities of this software to identify and monitor top-freezing processes during the casting phase. This key undertaking is in line with BFI’s efforts to improve process efficiency and product quality through innovative technological measures.

Using a simulation-based approach involving CFD simulations, a sensitivity study of influence of process parameters affecting the heat flow distribution in the mould was performed. Once the critical parameters were identified, they were subsequently used to develop and train a machine learning model to identify the occurrence of these events with the final goal of adaptation for an online system. For the AI-model, features were extracted from the time-series data for different casters at AGDH. The model was transferred to AGDH and steps have been undertaken to adapt and implement the model within the casting process.

Since the developed system was transferred at the end of the RealTimeCastSupport project, no online tests to predict the top-freezing events were performed during continuous casting. The next steps involve an offline-based fine-tuning of the model, to internal specifications. The One class SVM model showed the most promise to detect the top-freezing with the highest accuracy. At present the recall values are still too low. However, the approach is promising, and AGDH is currently training the model further with newer data over a longer time period. Furthermore, hyperparameter tuning of the one-class SVM parameters is also being performed. This will further allow an optimized system with increased recall and precision parameter, making it practically feasible.

## Discussion

### Reduction of surface defects on heavy plates

A carbon-rich layer near the strand surface can be formed or influenced by the casting powder or continuous casting process parameters. Subsequently, the carbon-rich layer can cause the formation of defects at the plate surface. To prevent the occurrence of surface defects, several measures have been taken to reduce the risk of carburization. In addition, comprehensive investigations by Eddy Current measurements figured out that almost all surface defects were detected after hot rolling.

Input variables from the continuous caster were evaluated by CFD simulations and afterwards used to develop and train an online system. The system was transferred to AGDH and connected to the existing database. The system will be finetuned offline to internal specifications. This will further allow an optimized system with increased recall and precision parameter.

## Conclusion

### Improvement of the product inspection procedure

It is possible to reduce the risk of surface defects by scarfing the slab surface. Scarfing each slab is an effective measure but is unlikely to be feasible for logistical and cost reasons. Therefore, AGDH performs eddy current measurements to detect surface defects after rolling. Once the support system can be online implemented into the current system, guided top-freezing controls can be performed. This offers an increased process safety due to the prediction of top-freezing and the potential to reduce disturbances in the mould.

### Benefits of real-time support system for advanced process control

During continuous casting, manual top-freezing controls must be carried out. Every manual performed mould control can affect the strand quality and even increase the risk of failure. Therefore, regular top-freezing controls are performed after a certain casting duration. However, top-freezing events between the regular controls cannot be detected and are a major risk for plant safety. The application of a real-time support system enables the prediction of top-freezing events during the whole casting process. Subsequently, this significantly increases the plant safety and offers to carry out top-freezing inspections in a more targeted manner in the future.

### Assessment of the chosen approach for further applications

In addition to top-freezing, several other defects can occur during continuous casting, such as transverse or longitudinal cracks. The developed real-time support system could be extended and trained with plant variables to predict the risk of crack formation.

A defect prediction, e.g. for longitudinal cracks, can also be a very important feature to be analysed in the future with the installed real-time support system.

The CFD-based digital twin of the casting machines delivers detailed insight of the flow and temperature fields as well as the thickness of the solidified shell. Since the Cu-mould is also included in the model, the local heat fluxes within the mould are also calculated, which can be compared to the online measurements of the mould cooling system. The parameter sensitivity study has revealed that the super heat of the melt in tundish, casting speed and inert gas injection at SEN has considerable influence on the computed temperature field at top surface of the steel melt thus the top-freezing phenomena. Although the CFD-based digital twin is developed for AGDH casters, it can be transferred to different plants (i.e., the production of different sizes, bloom or billet caster, etc.) without difficulty.

The implemented data-driven method is designed to receive and process continuously updated data from the continuous casting process. This dynamic exchange of information occurs at regular intervals, for example every 20 minutes, and triggers the execution of the model for analysis. The results of this analysis are then visually displayed as indicators.

If a high potential for a top-freezing event is detected, an immediate response is initiated, indicated by the illumination of a red lamp. Conversely, a green light is activated when conditions are considered satisfactory. Should the probability of a top-freezing event or normal conditions be similar, a yellow light will illuminate.

To ensure a comprehensive record of these events, all data is systematically stored in a dedicated database. Regular maintenance is ensured through scheduled updates; approximately once a month, the model is re-trained using the latest data. At this stage, parameters are fine-tuned to ensure optimal performance.

We are confident that the implementation of this sophisticated methodology will significantly improve the ability to identify top-freezing events.

## Ethics and consent

Ethical approval and consent were not required.

## Data Availability

More Data and information are given in the Periodic Reports of RealTimeCastSupport ^13^. Additional plant data is stored at AGDH but cannot be published for confidentiality reasons. We may not pass on or publish the data in order to rule out the possibility of conclusions being drawn about internal know-how or internal processes. In order to apply for access to the data and the conditions under which access will be granted please contact Robin Jentner ( robin.jentner@dillinger.biz) or Nico Neuber ( nico.neuber@dillinger.biz).
