# Supplementary material for: Embedded real-time analysis of continuous casting for machine-supported quality optimisation
Source: Open Res Eur. 2025 Nov 28;5:233. Originally published 2025 Aug 8. [Version 2] doi: 10.12688/openreseurope.20547.2 (PMC12848349; doi:10.12688/openreseurope.20547.2)
Supplement: Peer review history

# Reviewer report: Reviewer response for version 2

**DOI:** 10.21956/openreseurope.23702.r68111

**Published:** 2026-01-27

**Authors:** Youcheng Zong

**Affiliations:** 1 Northeastern University, Shenyang, Liaoning, China

I have reviewed the revised version of this manuscript and I am satisfied with the authors' responses to the previous comments. The clarity of the text has been significantly improved, specifically regarding the descriptions of the fluid mechanical conditions and the specific definitions of the casting strands.

 The paper presents a practical combination of CFD simulations and data-driven analysis (SVM) to address the top-freezing issue in an industrial setting. While I note that the CFD model uses some simplifications and the current recall rate of the detection system is relatively low, the authors have transparently acknowledged these limitations and outlined the necessary offline fine-tuning. For an applied engineering study, the current approach is valid and provides useful insights for process digitalization. Regarding the data availability, I acknowledge that full reproducibility is restricted due to the confidentiality of the industrial plant data, which is understandable. I have no further objections and recommend the article for approval.

Is the study design appropriate and does the work have academic merit?

Yes

Is the work clearly and accurately presented and does it cite the current literature?

Yes

If applicable, is the statistical analysis and its interpretation appropriate?

Yes

Are all the source data underlying the results available to ensure full reproducibility?

Partly

Are the conclusions drawn adequately supported by the results?

Yes

Are sufficient details of methods and analysis provided to allow replication by others?

Partly

Reviewer Expertise:

Steelmaking, data-driven process control, smart manufacturing.

I confirm that I have read this submission and believe that I have an appropriate level of expertise to confirm that it is of an acceptable scientific standard.

# Reviewer report: Reviewer response for version 2

**DOI:** 10.21956/openreseurope.23702.r65000

**Published:** 2025-12-30

**Authors:** Dheeraj Kumar Saini

**Affiliations:** 1 Microstructure Physics and alloy Design, Max-Planck-Gesellschaft, Munich, North Rhine Westphalia, Germany

The authors have only partially addressed the mentioned comment and have not explained the underlying physics of the problem. The revision also does not include the necessary information about the boundary conditions.

Is the study design appropriate and does the work have academic merit?

Partly

Is the work clearly and accurately presented and does it cite the current literature?

Partly

If applicable, is the statistical analysis and its interpretation appropriate?

No

Are all the source data underlying the results available to ensure full reproducibility?

No

Are the conclusions drawn adequately supported by the results?

Partly

Are sufficient details of methods and analysis provided to allow replication by others?

No

Reviewer Expertise:

Casting and solidification, Continuous casting

I confirm that I have read this submission and believe that I have an appropriate level of expertise to confirm that it is of an acceptable scientific standard, however I have significant reservations, as outlined above.

# Reviewer report: Reviewer response for version 2

**DOI:** 10.21956/openreseurope.23702.r64999

**Published:** 2025-12-30

**Authors:** Koulis Pericleous

**Affiliations:** 1 University of Greenwich, London, England, UK

This is a much improved version of the original submission  that addresses most of the points raised in my review. 

 I have no new comments to make and I am happy to approve the article.

Is the study design appropriate and does the work have academic merit?

Partly

Is the work clearly and accurately presented and does it cite the current literature?

Partly

If applicable, is the statistical analysis and its interpretation appropriate?

Not applicable

Are all the source data underlying the results available to ensure full reproducibility?

No

Are the conclusions drawn adequately supported by the results?

Partly

Are sufficient details of methods and analysis provided to allow replication by others?

No

Reviewer Expertise:

CFD expert specialising in metals processing including the continuous casting of steel plate.

I confirm that I have read this submission and believe that I have an appropriate level of expertise to confirm that it is of an acceptable scientific standard.

# Reviewer report: Reviewer response for version 1

**DOI:** 10.21956/openreseurope.22232.r59284

**Published:** 2025-09-25

**Authors:** Koulis Pericleous

**Affiliations:** 1 University of Greenwich, London, England, UK

**Title: **Embedded real-time analysis of continuous casting for machine-supported quality optimisation

 The project attempts to optimise mould control during continuous steel casting to prevent top-freezing (of powder or steel?) by replacing manual intervention with a real-time support system based on Big Data statistical analysis  and the development of a Digital Twin.

 The submitted report claims the following achievements:

 - Correlation of statistical results with surface defect detection

 However there is not enough information provided to satisfy this statement and data withheld due to commercial confidentiality.

 - A 3D Digital Twin of the CC process based on CFD software, accounting for heat transfer, solidification and inert gas feeding.

 In my assessment, the CFD model is not state of the art, with several important omissions which would have a bearing on the top surface temperature and freezing condition. The powder thickness is assumed constant and there is no accounting for melting. The flow generated by the argon injection is known to affect the slag layer  and therefore top surface heat loss.

 The commercial code ANSYS fluent has been used to model wall solidification , using a porosity approach with a fixed value resistance  obtained from Fluent. There have been over the years numerous CC models developed which can provide guidance (e.g. works by B.G Thomas,  

https://doi.org/10.1002/srin.201700312 ,  from *RWTH Aachen, etc.)*

 - Offline use of the Digital Twin to establish conditions for detected behaviour.

 This appears to be work in progress without evidence provided of successful linkup with the industrial partner - perhaps due to confidentiality

 In conclusion the project objective relies on the use of a CFD model Digital Twin , which in this case unfortunately does not represent the full physics of the process.

Is the study design appropriate and does the work have academic merit?

Partly

Is the work clearly and accurately presented and does it cite the current literature?

Partly

If applicable, is the statistical analysis and its interpretation appropriate?

Not applicable

Are all the source data underlying the results available to ensure full reproducibility?

No

Are the conclusions drawn adequately supported by the results?

Partly

Are sufficient details of methods and analysis provided to allow replication by others?

No

Reviewer Expertise:

CFD expert specialising in metals processing including the continuous casting of steel plate.

I confirm that I have read this submission and believe that I have an appropriate level of expertise to state that I do not consider it to be of an acceptable scientific standard, for reasons outlined above.

# Reviewer report: Reviewer response for version 1

**DOI:** 10.21956/openreseurope.22232.r58827

**Published:** 2025-09-01

**Authors:** Dheeraj Kumar Saini

**Affiliations:** 1 Microstructure Physics and alloy Design, Max-Planck-Gesellschaft, Munich, North Rhine Westphalia, Germany

**Technical Comments**

1. Please clarify what you mean by *“fluid mechanical condition”*, as mentioned repeatedly throughout the manuscript.
2. The introduction states that the thermal and fluid behavior in continuous casting (CC) is “roughly known.” This statement is inaccurate, since extensive literature exists on thermal–fluid flow multiphysics in the tundish and mold. For example, Prof. B. G. Thomas (Colorado School of Mines, USA), Prof. Deepak Majumdar (IIT Kanpur, India), and Prof. P. K. Jha (IIT Roorkee, India) have made significant contributions in this field. Please revise accordingly.
3. In the objective section, instead of vaguely referring to a large number of influencing parameters, explicitly list the parameters that affect the CC process.
4. Figure 1 requires a more detailed explanation in the text.
5. In Figure 2, clarify what the y-axis represents and explain the meaning of *“control”*.
6. Define what is meant by *“strand 1”* and *“strand 2.”*
7. Explain what *“SVM CC4”* and *“CC5”* refer to in the text.
8. Provide a clear discussion of the parameters and boundary conditions used in the CFD part of the study.
9. In Figures 6 and 8, the temperature scale appears to vary from -2 °C to 16 °C. If this refers to the steel flow field, it is physically inconsistent since the melting point of steel is around 1500 °C. Please clarify whether the temperature values have a different meaning here.
10. The manuscript lacks coherence. For instance, in one section, you mention controlling the top freezing of the steel melt with big data, while in another, you discuss modifications to the SEN and its submergence depth—without explaining the relationship between the two.

**Overview Comments**

1. Expand abbreviations such as *RFCS*, *NoSQL*, and *goDB* the first time they appear.
2. The *Results* part of the abstract should be rewritten for clarity and completeness.
3. The manuscript requires thorough language editing to correct grammar and improve readability.
4. In Figure 1, the font size and detailing are too small—please improve the clarity.
5. Use consistent temperature units throughout the manuscript.
6. The overall language quality is poor. The manuscript must be carefully proofread before submission.
7. Figure 9 is not legible and needs to be revised for clarity.

Is the study design appropriate and does the work have academic merit?

Partly

Is the work clearly and accurately presented and does it cite the current literature?

Partly

If applicable, is the statistical analysis and its interpretation appropriate?

No

Are all the source data underlying the results available to ensure full reproducibility?

No

Are the conclusions drawn adequately supported by the results?

Partly

Are sufficient details of methods and analysis provided to allow replication by others?

No

Reviewer Expertise:

Casting and solidification, Continuous casting

I confirm that I have read this submission and believe that I have an appropriate level of expertise to state that I do not consider it to be of an acceptable scientific standard, for reasons outlined above.
